# Study on the effect of cementation solution concentration on sand fixation by fiber reinforced MICP

**DOI:** 10.1371/journal.pone.0329673

**Published:** 2025-08-11

**Authors:** Huan Tao, Chaochao Sun, Jili Qu, Yuandong Huang

**Affiliations:** 1 College of Environment and Architecture, University of Shanghai for Science and Technology, Shanghai, China; 2 College of Civil Engineering, Kashi University, Kashi, Xinjiang, China; UNICAMP, University of Campinas, BRAZIL

## Abstract

This study systematically investigates the influence of cementation solution concentration on the sand fixation effect induced by palm fiber-enhanced microorganisms through microbial induced calcium carbonate precipitation (MICP), aiming to optimize its application in ecological restoration and engineering reinforcement. A series of experiments including unconfined compressive strength tests, direct shear tests, permeability tests, nuclear magnetic resonance analysis, calcium carbonate content determination, scanning electron microscopy (SEM), and X-ray diffraction (XRD) evaluates the mechanical properties, permeability, and microstructural characteristics of MICP-treated sand under varying cementation concentrations ranging from 0.2 to 0.7 mol/L. Results show that a concentration of 0.5 mol/L yields the best mechanical performance, with significantly higher unconfined compressive strength (666.65 kPa) and shear strength compared to other concentrations. At lower concentrations from 0.2 to 0.4 mol/L, increasing the concentration enhances calcium carbonate deposition, which improves mechanical properties and reduces both permeability coefficient and porosity. In contrast, higher concentrations above 0.5 mol/L inhibit microbial enzymatic activity, leading to reduced calcium carbonate content and mechanical strength, along with increased permeability and porosity. Microscopic analysis reveals that at 0.5 mol/L, calcium carbonate crystals form densely and uniformly, effectively filling pore spaces and strengthening inter-particle bonding. Therefore, 0.5 mol/L represents an optimal balance between performance and cost, reducing resource waste while ensuring mechanical enhancement and supporting applications in sand dune stabilization, windbreaks, sand fixation, and ecological vegetation restoration.

## Introduction

**Fig 1:**
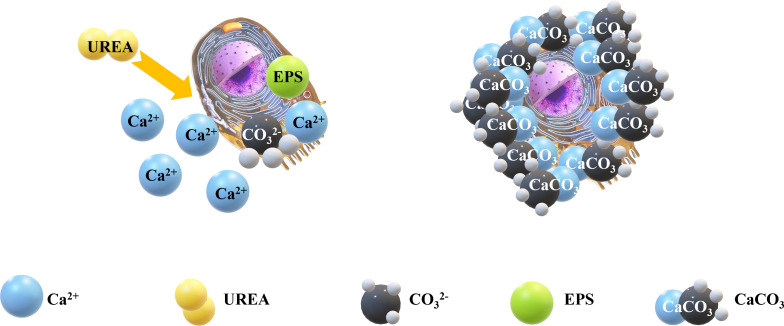
MICP mechanism diagram.


Microbial-induced calcium carbonate deposition (MICP) has emerged recently as an innovative technique for improving soil and environmental conditions within the fields of environmental and civil engineering. Owing to its advantages-such as energy efficiency, environmental friendliness, and minimal disturbance to soil structures-MICP has garnered significant attention from researchers both domestically and internationally. MICP technology primarily provides nutrients and calcium ions (Ca^2^⁺) solution. Calcium ions (Ca^2^⁺) play a critical role in the hydrolysis of urea by reacting with carbonate ions (CO_3_^2^⁻), which are produced during urea hydrolysis, to form insoluble calcium carbonate (CaCO_3_) precipitate. This precipitate serves as the primary mineralization product in the MICP process. Urea hydrolysis is enzymatically catalyzed by microorganisms, such as urease-producing bacteria, yielding carbonate ions (CO_3_^2^⁻) and ammonium ions (NH₄⁺), formula (1)-(2), Calcium ions (Ca^2^⁺) react with carbonate ions (CO_3_^2^⁻) to form insoluble calcium carbonate (CaCO₃) precipitates through a precipitation reaction, formula (3). Among these, carbonate ions combine with metal ions to form carbonate ions, and carbonate acts between sandy soils, enabling the sand to constitute a whole with a certain strength [1,2], to improve the properties of the soil, such as strength characteristics [3] and resistance to liquefaction [4]. The microbial curing process is influenced by numerous factors, including the concentration of Ca+, urease activity of the bacterial solution [5,6] water content [7], and environmental factors, which will exert an impact on the mechanical properties of the solidified sample [8,9]. The MICP reaction process diagram is presented in Fig 1.

$$CO{\left({NH}_{2}\right)}_{2}+H_{2}O\to C{O\;}_{2}\;+NH_{3}\;\mathrm{\backslash ]}$$ (1)

$$C{O\;}_{2}+NH_{3}\;+H_{2}O\to \;CO_{3}^{2-}+2NH_{4}^{+}\;\mathrm{\backslash ]}$$ (2)

$$Ca^{2+}+CO_{3}^{2-}\;\to CaCO_{3}\mathrm{\backslash ]}$$ (3)

The reinforcement effect of MICP is subject to numerous factors. A significant number of relevant studies have been carried out based on terrigenous sand, ranging from [10–15]. Furthermore, more extensive inquiries have been conducted regarding the enhancement of the treatment effect and efficiency of MICP for terrigenous sand, from [16–19]. Liu Hanlong et al. [20] indicated that MICP treatment can boost the dynamic characteristics of calcareous sand. Liu et al. [21] investigated the strength and microstructure of MICP-reinforced calcareous sand and ascertained that the strength and stiffness of the sample increased with the increase of calcite content, and the peak cohesion of the sample was in an exponential relationship with the number of times of cementation solution treatment. Zhu Jikang et al. [22] examined the addition of solid strength to MICP calcium sand by calcium source and found that the unconfined compressive strength of calcium chloride as the calcium source was approximately twice that of calcium acetate. Zheng Junjie et al. [23] determined that the skeletal difference of calc sand and the uniformity of calcium carbonate distribution were the main factors influencing the strength of calc sand. MICP can enhance the unconfined compressive strength of calcareous sand, which is a critical factor in the tensile failure of building foundations, differential settlement of airport pavements, and resistance to impact loads [24–26]. However, the inherent trade-off between strength and toughness remains a persistent challenge in the development of engineering materials [27]. MICP-solidified silica sand likewise encounters the identical issue. Despite its potential for high strength, MICP-treated material remains inherently brittle, with a tendency to experience abrupt strength loss upon failure-posing considerable safety concerns in engineering applications. To address this limitation, a novel approach combining fiber reinforcement with MICP has been proposed to improve the unconfined compressive strength and overall performance of calcareous sand. Lin et al. [28] discovered that fiber can diminish the brittleness and permeability of MICP-cured calcareous sand. The studies of Lei [29] and Fang [30] have indicated that the unconfined compressive strength of fiber-reinforced MICP-solidified calc sand can be conspicuously enhanced. Based on a series of splitting tensile tests, Consoli et al. [31] demonstrated that the combined effect of fiber and cement material can significantly elevate the tensile strength of soil. Li et al. and Choi et al. [32–34] integrated MICP technology with fiber reinforcement, and the unconfined compressive strength exceeded 2 MPa. LYU et al. [35] employed polypropylene fiber, basalt fiber, and carbon fiber in conjunction with MICP to improve the mechanical properties of silica sand, and its strength and toughness were markedly enhanced.

The combined application of MICP and fiber reinforcement technology demonstrates significant potential in improving the mechanical properties of sand, particularly in enhancing strength, toughness, and overall performance. This study systematically investigates the influence of cementation solution concentration on palm fiber-reinforced MICP-treated sand through comprehensive experiments, including unconfined compression tests, direct shear tests, permeability tests, nuclear magnetic resonance (NMR) analysis, calcium carbonate content measurements, scanning electron microscopy (SEM), and X-ray diffraction (XRD). The results reveal that a cementation solution concentration of 0.5 mol/L optimally balances cost and efficiency, preventing resource wastage while ensuring sufficient mechanical enhancement. This concentration not only promotes rapid crust formation on sand dunes, mitigating wind erosion and facilitating vegetation restoration, but also significantly improves the shear strength and unconfined compressive strength of solidified sand, making it suitable for applications such as sand barrier construction, slope stabilization, and desert highway reinforcement. Additionally, permeability and porosity analyses indicate that the 0.5 mol/L group reduces water evaporation while maintaining rainwater infiltration, supporting water-retaining planting and root development. The study further highlights the critical role of calcium carbonate (CaCO₃) as the primary cementing agent in MICP, providing practical insights for optimizing sandy soil consolidation and ecological restoration in arid environments.

## Experimental material

### Siliceous sand

**Fig 2:**
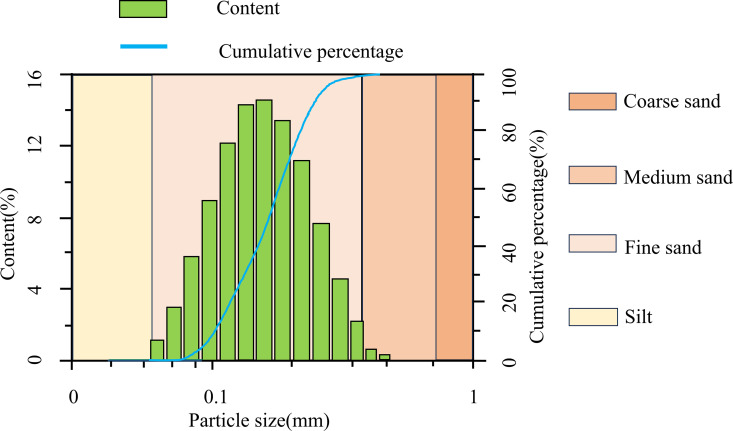
Size distribution diagram of sand particles.

The sand employed in the test is siliceous sand from the Kashi area. (Given the limited scale of collection and the non-commercial nature of this scientific research activity, no additional permission is required, as per the relevant legal exemption provisions.) The particle size gradation is depicted in Fig 2, and the detailed physical and mechanical properties are presented in Table 1. In accordance with the “Engineering Classification Standard of Soil” [36] the sand is classified as poorly graded.

**Table 1:** Physical and mechanical properties of sand.

Name	Moisture content (%)	Dry density (g/cm^3^)	Void ratio	Gravity	Effective particle size_10_	C_U_	C_C_	PH value
Desert soil	0.7	1.58	0.758	2.68	0.086	2.18	1.06	8.76

### Palm fiber

**Fig 3:**
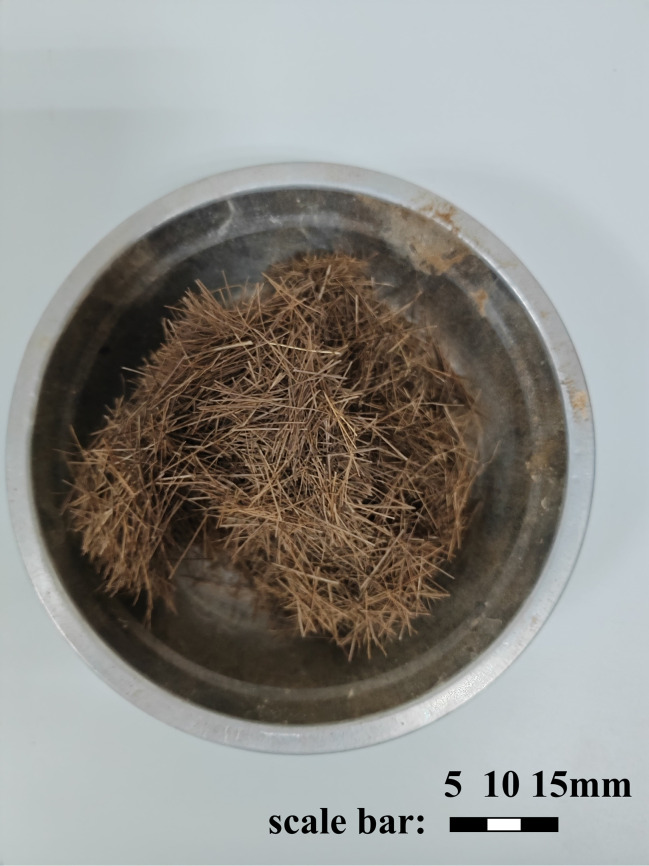
Palm fiber.

The fiber utilized in the test is palm fiber (Fig 3), and its physical and mechanical properties are disclosed in Table 2. The palm fiber employed in this study is sourced from palm trees cultivated in Yunnan Province, China. The fiber was procured from Xinjiang Xingtai Fiber Technology Co., Ltd.

**Table 2:** The physical and mechanical properties of palm fiber.

Palm fiber density (g/cm^3^)	Calorific value (Kcal/kg)	Moisture (%)	Diameter (µm)	Ash content (%)	Impurity content (%)	Modulus of elasticity (MPa)	Tensile strength (MPa)	Elongation at break (%)
1.28	4000	12-30	0.2-0.3	3	0.8	800-1900	87-166	19.0-21.0

Pre-treatment and Mixing Protocol for Palm Fiber-Reinforced Sand Samples

1 Fiber Pre-treatment

To ensure uniform diindspersion and optimal performance of palm fibers within the sand matrix, the following pre-treatment steps are performed:

Cutting: Palm fibers are weighed according to the specified dosage and cut to a predetermined uniform length to facilitate consistent distribution.

Cleaning and Drying: Fibers are thoroughly washed to remove surface impurities and natural oils, followed by drying. This process enhances the cleanliness of the fiber surface and improves the interfacial adhesion between the fibers and the surrounding sand or soil particles.

2 Mechanical Mixing Procedure

Initial Moistening: The sand or soil is pre-moistened prior to fiber addition. This step reduces fiber entanglement and facilitates more uniform dispersion throughout the matrix.

Stirring Conditions: Fibers are gradually introduced into the moistened sand, and low-speed mechanical stirring is employed to ensure even distribution. Continuous and consistent mixing promotes effective contact between fibers and particles.

Batch Addition: Both sand and fibers are introduced in multiple increments rather than all at once, which minimizes fiber agglomeration and improves the homogeneity of the mixture.

Mixing Duration: Adequate mixing time is critical. Insufficient mixing can result in poor uniformity, while excessive mixing may lead to fiber breakage. Based on the experimental conditions in this study, an optimal mechanical stirring time of approximately 5 minutes was determined to achieve a well-mixed, uniform composite.

Calorific Value and Organic Matter Content:

The calorific value of palm fiber is positively correlated with the concentration of organic constituents such as cellulose, hemicellulose, and lignin. These components can serve as carbon and energy sources for microorganisms (e.g., urease-producing bacteria), thereby indirectly influencing the efficiency of MICP.

Microbial Metabolic Support:

During the gradual degradation of palm fibers in sandy soil, the released organic compounds may partially fulfill the metabolic requirements of microorganisms, promoting their growth and urease activity. This enhanced enzymatic activity accelerates the hydrolysis of urea, increasing the availability of CO_3_^2^ ⁻ ions and consequently facilitating calcium carbonate precipitation.

Stability of High-Calorific-Value Fibers:

Fibers with higher calorific values typically exhibit a greater degree of lignification, which reduces their biodegradation rate. This property contributes to the long-term structural stability of MICP-treated sandy soils by maintaining fiber integrity over extended periods.

The incorporation of 12 mm palm fibers significantly enhances the mechanical properties of MICP-treated sand by forming a stable reinforcement network that reduces crack propagation and improves overall toughness. This fiber length facilitates uniform dispersion within the matrix and promotes effective microbial adhesion, thereby optimizing calcium carbonate precipitation. A low fiber content of 0.15% ensures adequate reinforcement while maintaining sufficient permeability, allowing for efficient diffusion of microorganisms and nutrients throughout the soil matrix. This balanced combination enhances both the mechanical performance and the bio-cementation efficiency of the MICP process. As reported in studies [37,38], the use of 12 mm palm fibers at a dosage of 0.15% effectively prevents fiber agglomeration, maintains structural integrity, and supports microbial activity, making it a promising strategy for soil stabilization applications. Due to the fact that the palm fiber length of 12 mm exerts the most favorable impact on the peak stress and residual stress of quartz sand, the palm fiber length of 12 mm [37], the optimal fiber content is 0.15% [27].

### Bacterial culture

**Fig 4:**
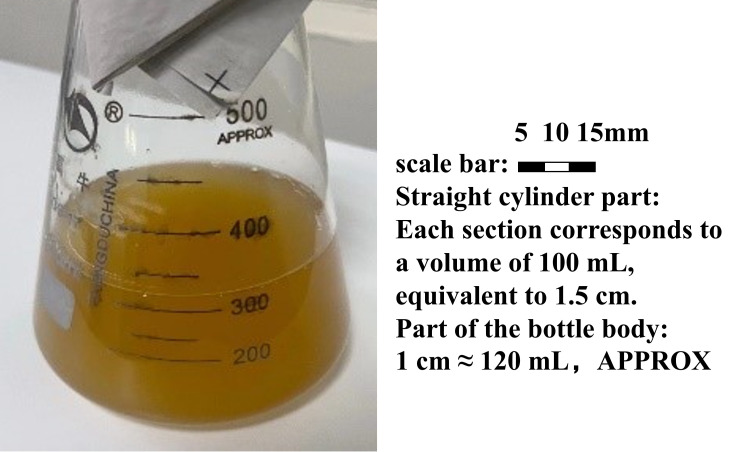
Sporosarcina pasteurii.

**Fig 5:**
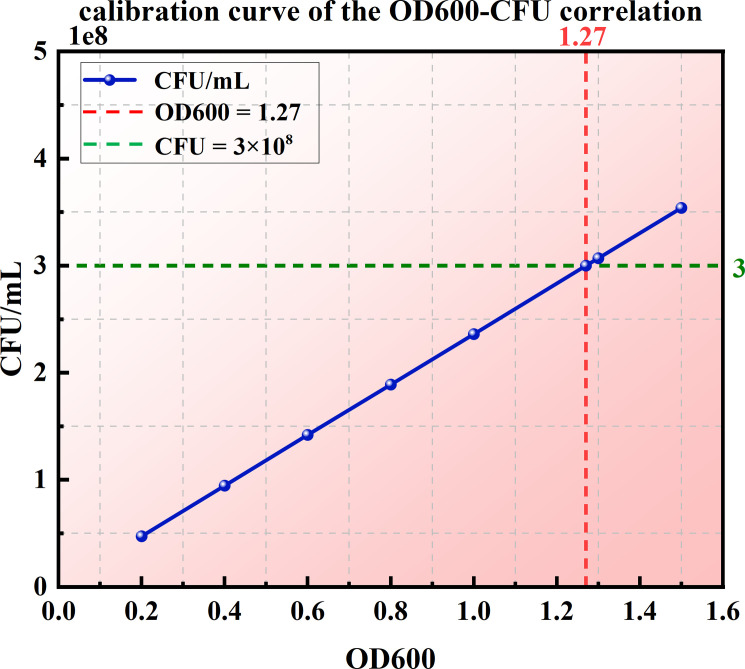
Calibration curve of the correlation between OD600 and CFU.

*Sporosarcina pasteurii* (Fig 4) was adopted in the experiment. The strain was obtained from the U.S. Culture Preservation Center, and ATCC 1376 NH4-YE solution medium (containing 20 g yeast extract per liter was employed. 15.73 g Trisbase, 10 g (NH_4_)_2_SO_4_, pH 9.0) were utilized for activating the culture. The bacteria should be sterilized at 120°C for 30 min prior to inoculation and then activated for 24 hours in a constant-temperature oscillating incubator with a rotational speed of 120 rpm/min and a temperature of 30°C. The urease activity of the bacterial solution was 3.11 mM·urea hydrolysed/min as determined by the conductivity meter. The OD600 of the bacterial solution tested by the enzyme-labeled instrument was 1.27 (corresponding to 3 × 10^8^ (CFU) mL^-1^). The calibration curve depicting the correlation between OD600 and CFU is in agreement with the data range reported in the references [39–41], as illustrated in Fig 5.

### Cementation solution

The cementation solution utilized in the test was a blend of calcium chloride (CaCl_2_) and urea with an equivalent molar concentration. CaCl_2_ furnishes the calcium source and urea provides the nitrogen source. The ratio of bacterial solution to cementation solution was determined as 1:3. A total of six groups of cementation solution with varying concentrations were constituted [38], namely 0.2, 0.3, 0.4, 0.5, 0.6, and 0.7 mol/L.

## Physical and mechanical properties test

### Unconfined compressive strength test

#### Sample cementation preparation.

**Fig 6:**
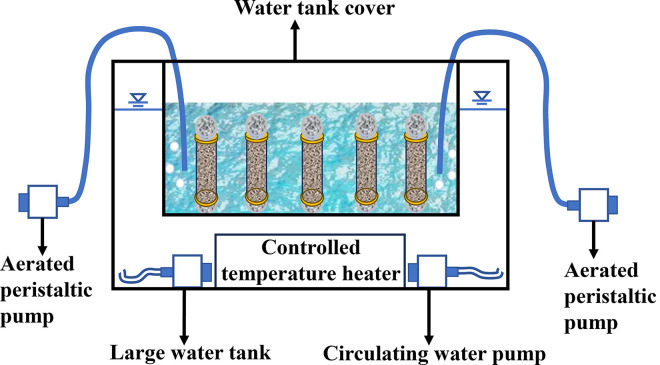
Schematic diagram of sample preparation for unconfined compressive strength test.

During the test, a cylindrical drilling mold was utilized for loading the specimens (with a height of 8 cm, a diameter of 3.91 cm, a diameter of 2 mm, and wrapped with gauze). Silica sand and palm fiber were homogeneously mixed in accordance with the preset fiber content, followed by the loading of samples. There were three parallel samples in each group, and the dry density was controlled at 1.26 g/cm^3^. All the sand columns were immersed in the bacterial solution for 2 hours [42–45]. After the colonization of bacteria, the samples were transferred to the curing box containing the cementation solution, where the cementation solution level was 3 cm higher than the top surface of the sample during the curing process (Fig 6). An air pump was employed to continuously inject air into the cementation solution, and after maintaining a constant temperature at 30°C for 7 days, the sample was inverted and air-dried to the natural moisture content (approximately 4%). After the curing process, the mold was removed, and the sample was rinsed with water and dried by natural air.

Unconfined compressive strength testing machine, meeting standard requirements [46], the unconfined compression test apparatus utilized was the ETM-205D microcomputer-controlled electronic universal testing machine, fabricated by Shenzhen Wanyi Test Equipment Co., LTD., featuring a maximum pressure of 200KN. A cylindrical sample mold with a diameter of 39.1 mm and a height of 80 mm was utilized for sample preparation. An electronic balance with an accuracy of 0.01 g was employed to ensure precise mass measurements. A constant temperature and humidity chamber, maintaining a controlled temperature of and a relative humidity of 95, was employed to regulate the environmental conditions during the curing process. The test soil sample was mixed uniformly in accordance with Standard [46]. Subsequently, the sample was compacted or molded into the cylindrical mold using standard compaction methods to achieve uniform density and minimize the presence of significant pores. After demolding, the sample was cured in the constant temperature and humidity chamber for 24 hours.

The sample was then positioned between the pressure plates of the testing machine, ensuring that its axis was precisely perpendicular to the pressure plates. Axial stress was applied at a constant strain rate of 0.5% per minute until failure occurred. The maximum failure load was recorded, and the unconfined compressive strength value was subsequently calculated based on the recorded data. The axial compression velocity of the specimen was set at 2 mm/min as a result of the fact that the palm fiber would markedly enhance the strength of the sandy soil. The axial strain was uniformly regulated at 22%, and the unconfined compressive strength of the specimen was computed in accordance with the formula:


$$\sigma =\frac{F}{A}\;$$ (4)

Where: A —cross-sectional area of the test block (mm^2^);

σ — unconfined compressive strength (kPa);

F — is the failure load (N).

Test at least three samples under the same conditions, calculate the mean and standard deviation. Exclude outliers to ensure data reliability.

### Direct shear test

#### Sample cementation preparation.

**Fig 7:**
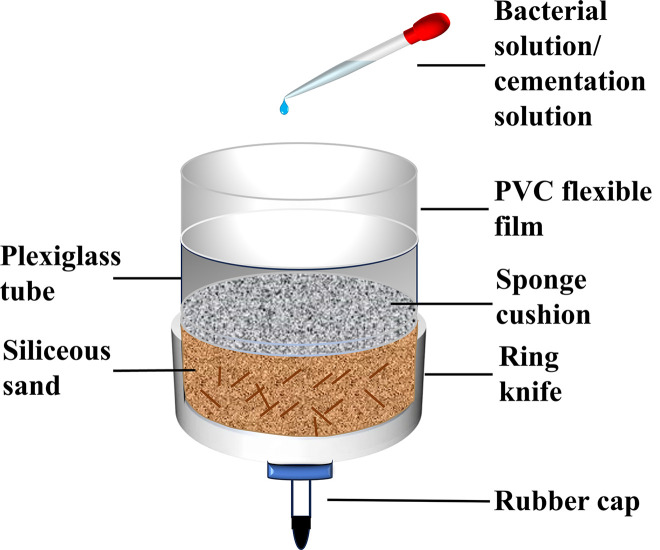
Schematic diagram of sample preparation for shear test.

The palm fiber is uniformly mixed with silica sand three times in accordance with the pre-determined proportion. Subsequently, it is loaded into the mold (with a diameter of 6.18 cm and a height of 2 cm) and compacted layer by layer (Fig 7). The rubber cap at the bottom of the device is removed, and the bacterial solution is gradually introduced from the top of the device to eliminate the air within the pores of the sample. Concurrently, a small amount of bacteria can adhere to the surface of the siliceous sand particles. The addition of the bacterial solution is ceased when it completely submerges the sample, and bacterial colonization is achieved after 2 hours. The rubber cap is opened, and the bacterial solution is drained from the bottom of the device. After the bacterial solution is drained, the rubber cap is covered and the cementation solution is injected, which is 3 cm higher than the sample to ensure that the bacteria and the solution can fully react within the sample. After 7 days of constant-temperature curing at 30°C, the waste solution is discharged from the bottom of the device. Once the cementing is completed, water is poured through the top of the unit to cleanse the soluble salt remaining in the sample, and then the sample is dried in the oven at 70°C until its quality remains stable.

The instrument utilized for the direct shear test is the NT.1JD-1 strain-controlled direct shear instrument fabricated by Nanjing Nantu Instrument and Equipment Co., LTD.. The vertical pressure $\sigma_{n}$ is set at 100 kPa, 200 kPa, and 300 kPa. The test was carried out according to the geotechnical test method standard [46]. The shear stress of the sample is calculated according to the following formula:


$$\tau =\frac{CR}{A_{0}}\times 10$$ (5)

Where: τ—Shearing stress (kPa);

C—Calibration coefficient of dynamometer (N/0.01 mm);

R—Dynamometer reading (0.01 mm);

A_0_—The initial area of the specimen (cm^2^).

### Penetration test

**Fig 8:**
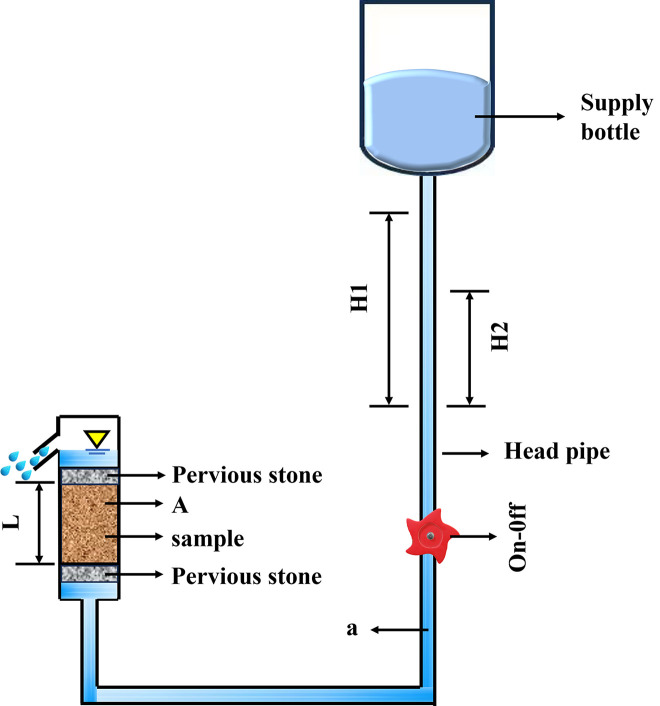
Penetration device diagram.

In penetration tests of MICP-reinforced fiber samples, experiments are typically performed under variable-head conditions to more accurately cementation solution flow through porous media. The sample’s preparation process relates to that of the unconfined compressive strength sample. The permeability test is executed on the cured aeolian sand sample in accordance with the geotechnical test method standard [47–49]. During the test, the permeability coefficient K is determined by the variable head method. The schematic diagram of the permeability device is displayed in Fig 8.

A variable-head permeability tester, flowmeter, water level gauge, and timer were employed. The preparation and curing procedures for the soil samples followed the same protocols as those used for the unconfined compressive strength test specimens. The soil sample was securely mounted in the permeameter to ensure complete sealing and prevent leakage. The hydraulic head difference was established by adjusting the water supply through the water head control device. The initial water head height and start time were recorded, and the timer was initiated accordingly. Continuous monitoring of water level changes and measurement of flow rates were conducted throughout the experiment. Upon achieving steady-state seepage conditions, all pertinent parameters were meticulously documented. Finally, the permeability coefficient was calculated using the variable-head method [46].


$$K=\frac{QL}{Aht}$$ (6)

Where: L—Infiltration diameter is equal to the sample height (cm);

A—Penetration section area (cm^2^);

t—Time (s);

Q—Infiltration capacity (cm^3^);

h—Water level difference (cm).

### Nuclear magnetic resonance test

**Fig 9:**
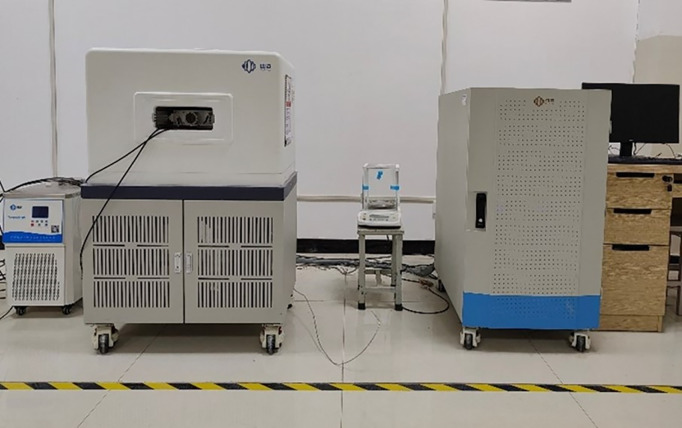
High precision NMR microanalyzer.

In line with the preparation process of the unconfined compressive strength sample, the sample was uniformly sliced into test blocks with a height of 8 mm, and the porosity was determined. The test instrument (Fig 9) employed was a high-precision nuclear magnetic resonance micro-analyzer manufactured by Suzhou Niumar Instrument.

### Calcium carbonate content measurement

The calcium carbonate content in the sand column test samples shielded by gummy solutions of varying concentrations was measured independently. The test procedure was as follows: The center of the sand column sample was divided into 20 g samples, rinsed anew with ultra-pure water, and subsequently air-dried. An excess of 0.1 mol/L dilute hydrochloric acid was added to each sample for reaction. Once no bubbles emerged from the samples, the residue was filtered, rinsed, and air-dried with ultra-pure water, and weighed as m. Subsequently, the calcium carbonate content of each sample was calculated as n:


$$n=\frac{20-m}{20}\times 100\;\%\;$$ (7)

### Analytical methods

The SEM instrument is denominated Cold Field-Emission Scanning Electron Microscope, and its manufacturer and model number is Japan Hitachi, Regulus 8100. The EDS instrument is styled as Energy Dispersive Spectrometer, and its manufacturer and model number is Bruker, QUANTAX EDS. The XRD instrument is termed X-ray Diffractometer, and its manufacturer and model number is Japan, Rigaku D/max2500. All of them originate from Jiantu Technology (Suzhou) Co., LTD.

### Schematic flowchart

**Fig 10:**
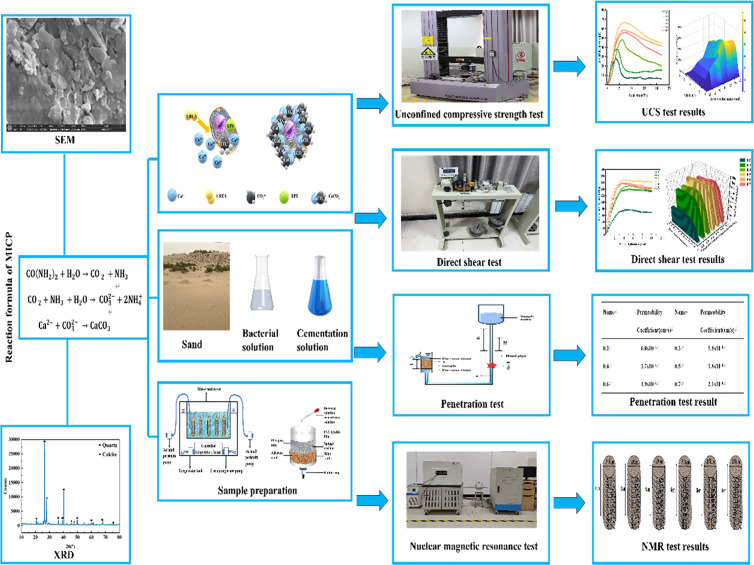
Schematic flowchart.

The Schematic flowchart is illustrated in Fig 10.

## Results and discussion

### Unconfined compressive strength test results analysis

**Fig 11:**
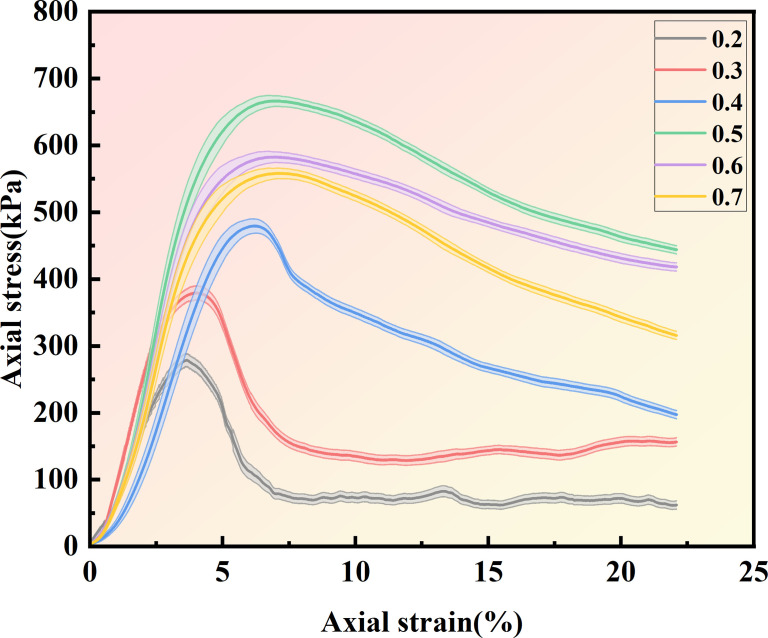
Stress-strain curves of 6 samples with different cementation solution concentrations.

The stress-strain curve was derived based on the unconfined compressive strength test, as presented in Fig 11. It is observable that the stress-strain curves of all samples exhibit typical strain-softening traits: initially, the stress escalates gradually with the increase of strain until it attains a peak value, and subsequently declines as the strain keeps increasing. When the concentration of the cementation solution is 0.5 mol/L, the maximum peak stress reaches 666.65 kPa. When the concentration of the cementation solution is 0.2 mol/L, the lowest peak stress is 278.59 kPa; when the concentration of the cementation solution is 0.4 mol/L, the peak stress is 479.41 kPa; when the concentration of the cementation solution is 0.6 mol/L, the peak stress is 582.63kPa. When the concentration of the cementation solution is 0.7 mol/L, the peak stress is 558.12 kPa. When the concentration of the cementation solution is 0.3 mol/L, the peak stress is 379.46 kPa This is because when the concentration of the cementation solution ascends from 0 to 0.4 mol/L, the content of the calcium source requisite for the curing reaction progressively augments to guarantee that the bacterial solution and the cementation solution can fully react, thereby augmenting the precipitation of calcium carbonate and reinforcing cementation. Nevertheless, when the concentration of the cementation solution continues to escalate to 0.5 mol/L, the concentration of calcium ions will result in the intensification of the alkaline environment, thereby impeding the normal metabolic activities of bacteria and inhibiting the generation of urea hydrolase [50,51]. Hence, the enhancing effect of MICP is the most pronounced when the concentration of the cementation solution is 0.5 mol/L. The reaction between the cementation solution and the bacterial solution at a concentration of 0.5 mol/L is extremely rapid, which renders the sample stronger and less susceptible to be destroyed initially.

**Fig 12:**
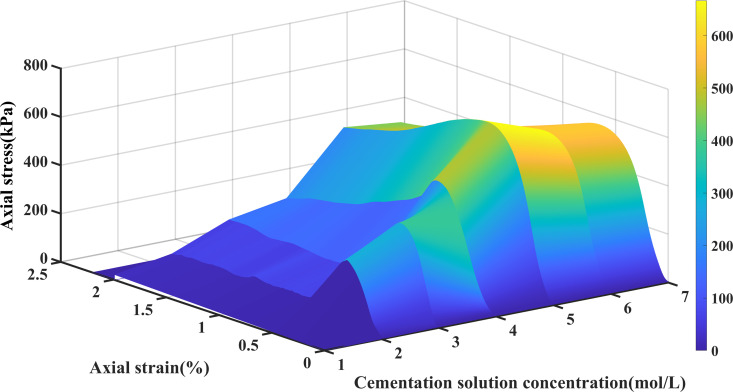
Three-dimensional stress-strain diagram of 6 samples with different cementation solution concentrations.

It can be observed from Table 3 that within the concentration range of 0.2–0.4 mol/L, the peak stress increases as the cementation solution concentration rises, while the standard deviation exhibits a marginal decrease. This trend indicates a progressive enhancement in cementation strength; however, the structural stability of the sample has not yet reached its optimal state. At 0.5 mol/L, the sample demonstrates peak intensity and standard deviation (3%), suggesting that this concentration achieves the most effective cementation and optimal microbial activity. At higher concentrations (0.6–0.7 mol/L), although the peak intensity remains relatively high, it begins to decline, with an increase in the standard deviation. This observation implies that an excessively high concentration of calcium ions may inhibit microbial enzyme activity or lead to non-uniform crystal deposition, thereby affecting the uniformity and stability of the cemented structure. Fig 12. illustrates that the stress-strain curve of the sample can be categorized into three distinct stages. In the elastic-plastic deformation stage, the stress-strain relationship displays a linear trend, and microcracks begin to develop in structurally weaker zones. As the deformation advances into the failure stage, the stress reaches its peak, during which the propagation of cracks results in the breakdown of cementation between sand and soil particles, eventually leading to their separation and culminating in complete failure. In the subsequent stress reduction stage, the specimen loses its structural integrity as a result of accumulated damage, and the stress declines rapidly with continued strain. This stress-strain behavior is closely linked to the curing conditions of the cementation solution. At low concentrations, the curing reaction proceeds slowly, preventing complete cementation within the allotted curing period and resulting in reduced unconfined compressive strength. In contrast, a moderate concentration facilitates an optimal reaction rate, allowing for sufficient and uniform cementation, thereby enhancing the unconfined compressive strength. However, a high concentration of the cementation solution, while accelerating the reaction with the bacterial solution, can inhibit urease activity, ultimately impeding the biomineralization process and compromising cementation efficiency. The curing reaction of the sample is inadequate, and the overall degree of cementation is low and uneven, resulting in low unconfined compressive strength, which is consistent with the conclusions of previous studies [52–54].

**Table 3:** Standard deviation (SD) of unconfined compressive strength corresponding to each concentration of cementation solution.

Cementation solution concentration (mol/L)	Average peak stress (kPa)	Standard deviation (kPa)
0.2	278.59	23.72
0.3	379.46	19.87
0.4	479.41	17.97
0.5	666.65	13.25
0.6	582.63	15.22
0.7	558.12	16.67

The concentration of the cementation solution is a critical factor influencing the consolidation strength of MICP. The relationship between cementation solution concentration and strength exhibits a significant nonlinear pattern. A moderate concentration facilitates effective cementation and enhances strength, whereas excessively high concentrations may suppress microbial activity or induce crystal defects, thereby hindering strength improvement. Under the experimental conditions described in this study, when the cementation solution concentration reaches 0.5 mol/L, the strength tends to stabilize and subsequently decline. As the concentration of the cementation solution increases from low to moderate levels (e.g., 0.2 to 0.5 mol/L), the elevated calcium ion concentration promotes the deposition of more calcium carbonate crystals, enhancing inter-particle cementation within sand and soil matrices and significantly increasing unconfined compressive strength. However, when the cementation solution concentration exceeds 0.5 mol/L, the strength growth typically stabilizes or decreases. This phenomenon can be attributed primarily to the inhibitory effect of high calcium ion concentrations on microbial enzymatic activity, particularly urease activity, which reduces the efficiency of carbonate deposition. Additionally, high-concentration cementation solutions may result in uneven crystal growth, forming larger but looser crystal structures that compromise the compactness and mechanical strength of the cemented material. Consequently, the influence of cementation solution concentration follows an “increasing then decreasing” or “peak” trend, with an optimal concentration point (0.5 mol/L in this experiment) at which maximum strength is achieved. Beyond this optimal concentration, the combined effects of microbial activity suppression and altered crystal structure lead to stagnation or reduction in strength.

### Direct shear test results analysis

**Fig 13:**
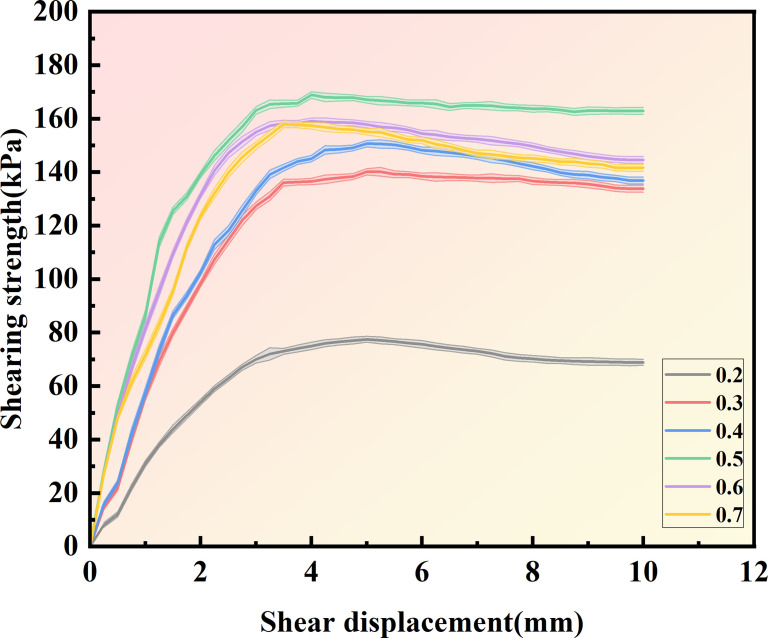
Shear displacement-shear stress curves of 6 different cementation solution concentrations ${\;(\sigma }_{n}=100Kpa)$.

**Fig 14:**
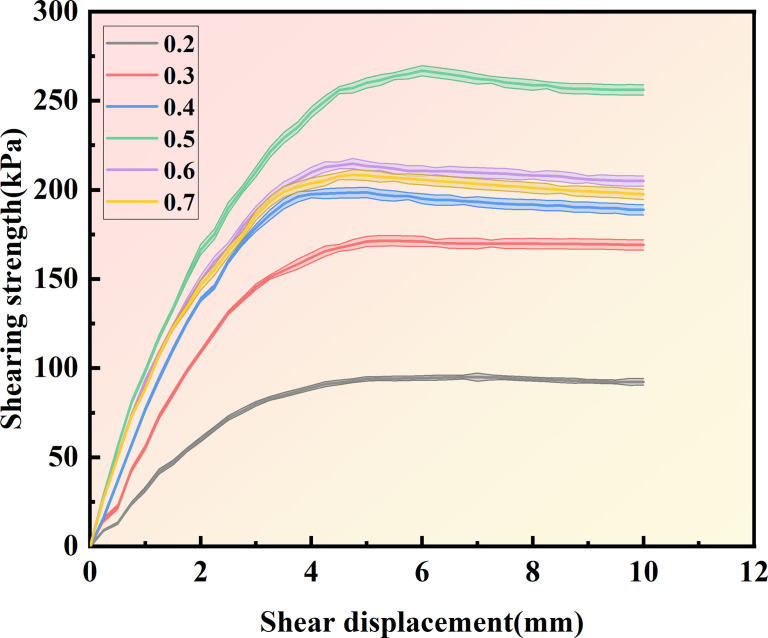
Shear displacement-shear stress curves of 6 different cementation solution concentrations ${\;(\sigma }_{n}=200Kpa)$.

**Fig 15:**
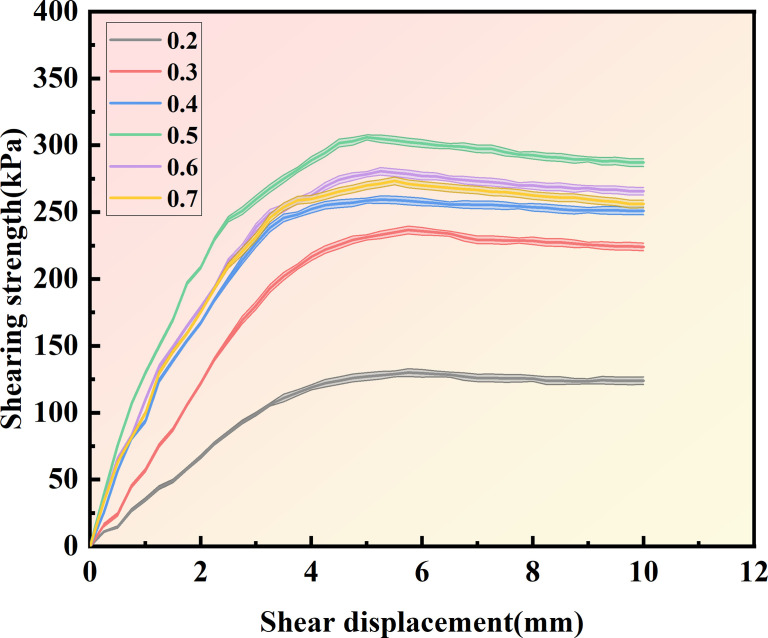
Shear displacement-shear stress curves of 6 different cementation solution concentrations ${\;(\sigma }_{n}=300Kpa)$.

Figs 13–15 show the shear displacement-shear stress curves of samples with six different cementation solution concentrations. When the vertical pressure σ_n_ is 100kPakPa, 200 kPa, and 300 kPa, the sample with a 0.5 mol/L cementation solution concentration exhibits the highest strength. The peak shear stresses are 168.85 kPa, 266.84 kPa, and 305.94 kPa, respectively. When the cementation solution concentration is 0.2 mol/L, the shear stress peaks are the lowest, being 77.5 kPa, 95 kPa, and 130 kPa, respectively. When the cementation solution concentration is 0.3 mol/L, the shear stress peaks are the lowest, being 140.14 kPa, 171.44kPa, and 236.6 kPa, respectively. When the cementation solution concentration is 0.4 mol/L, the shear stress peaks are 150.64 kPa, 198.56 kPa, and 259.35 kPa, respectively. When the cementation solution concentration is 0.6 mol/L, the shear stress peaks of the sample are 157.91 kPa, 208.48 kPa, and 273.38 kPa, respectively. When the cementation solution concentration is 0.7 mol/L, the shear stress peaks are 159.06 kPa, 214.77 kPa, and 280.5 kPa, respectively.

**Fig 16:**
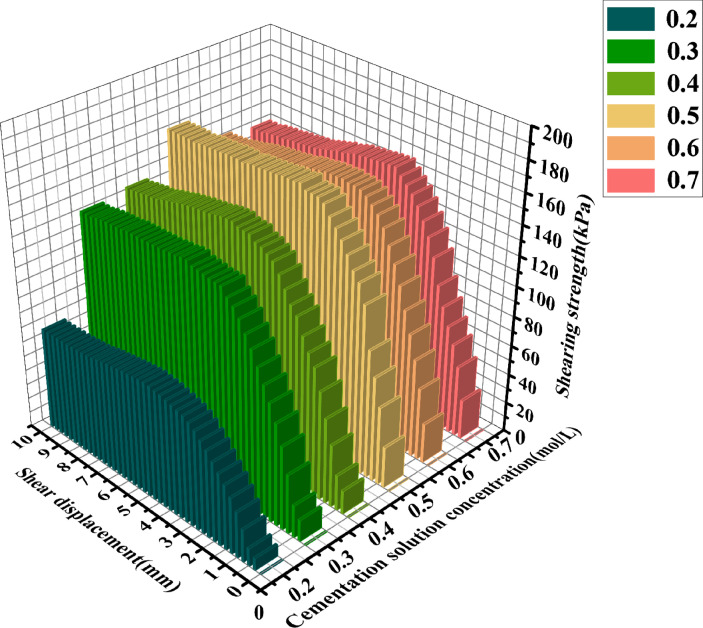
Three-dimensional graphs of shear displacement-shear stress of 6 different cementation solution concentrations ${\;(\sigma }_{n}=100Kpa)$.

**Fig 17:**
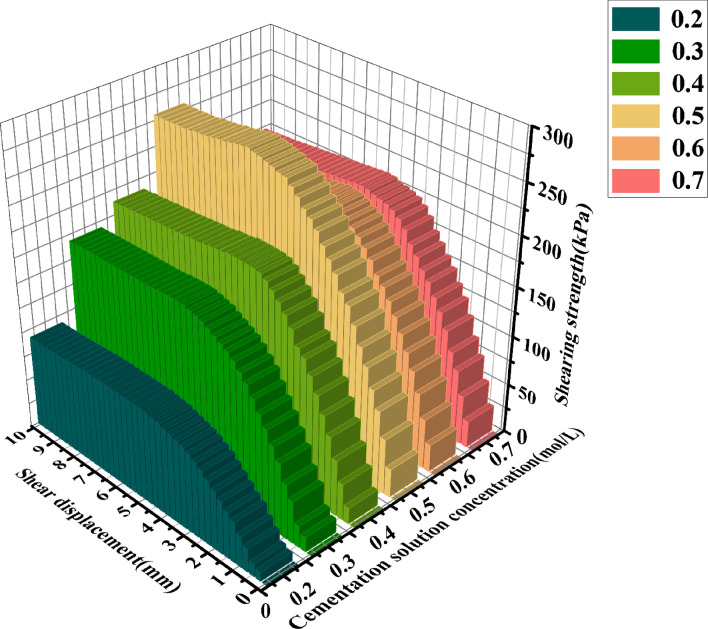
Three-dimensional graphs of shear displacement-shear stress of 6 different cementation solution concentrations ${(\sigma }_{n}=200Kpa)$.

**Fig 18:**
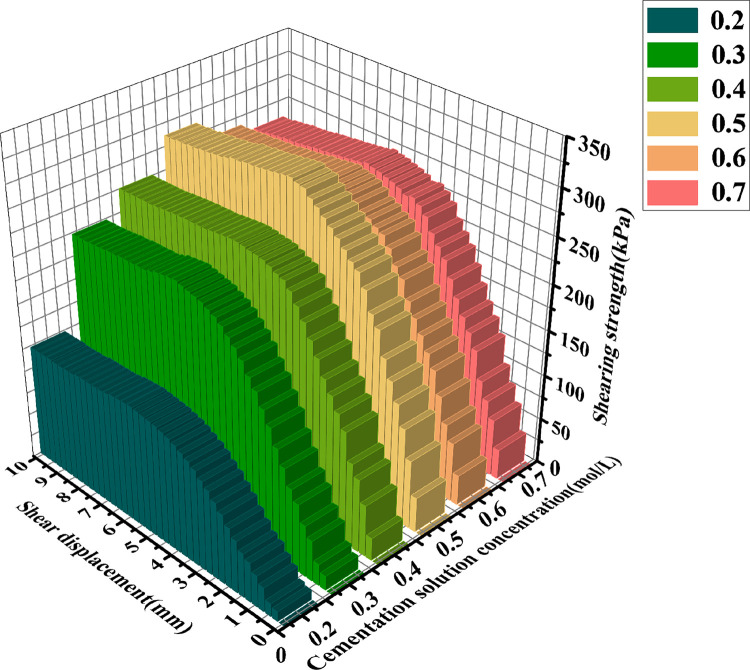
Three-dimensional graphs of shear displacement-shear stress of 6 different cementation solution concentrations ${\;(\sigma }_{n}=300Kpa)$.

It can be observed from Table 4 that under varying stress conditions, the standard deviation of shear stress is consistently reported. When the concentration of the cement solution is 0.5 moles, the standard deviation is minimal, and the shear stress reaches its maximum value, indicating a stable curing effect at this concentration. Within the concentration range of 0.2 to 0.4 moles, the standard deviation progressively decreases, while the shear stress correspondingly increases, suggesting an increase in calcium carbonate crystal content and enhanced sample curing. Conversely, when the concentration of the cement solution exceeds 0.6 moles, the standard deviation gradually increases, and the shear stress progressively decreases, indicating inhibited urease activity and a consequent reduction in calcium carbonate crystal content.After the shear stress attains its maximum value, the strength gradually diminishes with the increase in shear strain. As can be observed from Figs 16–18, when the shear displacement does not reach 5 mm, the shear stress of the six samples rises more rapidly when the concentration of the cementation solution is 0.5 mol/L, while the shear stress rises more slowly when the concentration of the cementation solution is 0.2 mol/L. As shear displacement increases in samples treated with six different cementation solution concentrations, the shear stress exhibits an immediate post-peak softening behavior. This phenomenon can be attributed to the dual effects of cementation solution concentration on the biocementation process. At low concentrations, the limited calcium availability restricts the bacterial curing efficiency, resulting in slower reaction kinetics and reduced calcium carbonate (CaCO₃) precipitation. Conversely, high-concentration solutions exhibit excessive alkalinity as a result of urea hydrolysis, which inhibits microbial urease activity and consequently impairs the curing reaction rate. An optimal cementation solution concentration promotes the efficient coupling of bacterially derived carbonate ions with available calcium sources, thereby maximizing CaCO₃ precipitation. These precipitated crystals enhance interparticle bonding, ultimately improving the soil’s shear strength.

**Table 4:** The standard deviation (SD) of shear stress corresponding to each concentration of cementation solution.

Cementation solution concentration (mol/L)	σ_n_ kPa	Shear stress peaks (kPa)	Standard deviation (kPa)
0.2	100	77.5	17.8
0.2	200	95	17.7
0.2	300	130	17.5
0.3	100	140.14	15.13
0.3	200	171.44	15.26
0.3	300	236.6	15.49
0.4	100	150.64	14.08
0.4	200	198.56	14.93
0.4	300	259.35	13.93
0.5	100	168.85	11.07
0.5	200	266.84	11.47
0.5	300	305.94	10.87
0.6	100	157.91	12.79
0.6	200	208.48	12.17
0.6	300	273.38	12.3
0.7	100	159.06	13.32
0.7	200	214.77	13.1
0.7	300	280.5	12.9

To investigate the impact of high-concentration cementation solution on bacterial activity, we examined the inhibitory effects of high-concentration cementation solutions (0.6 and 0.7 moles) on the urease activity of *Sporosarcina pasteurii*. Assuming that the urease activity in the control group (with a 0.5 mole concentration of cementation solution) is normalized to 100%, the data obtained under the experimental conditions explored in this study are presented in Table 5 below:

**Table 5:** Urease activity in high-concentration cementation solution.

Cementation solution concentration (mol/L)	Urease activity (U/mL)	Relative activity (%, with 0.5M as the control)
0.5 (Comparison)	15.2	100
0.6	10.8	71
0.7	6.5	43

1. Urease activity unit (U/mL): It is typically defined as the amount of enzyme required to hydrolyze urea and produce 1 μmol of NH₃ per minute under standard conditions.
2. Suppression Trends: At a concentration of 0.6 M, urease activity decreased by approximately 29%, and at 0.7 M, it decreased by 57%. These results indicate that as the concentration increases, the degree of inhibition becomes more pronounced. Urease activity exhibits a progressively decreasing trend with increasing concentrations of the cementation solution.

As can be observed from Table 5, This phenomenon can be attributed to the following mechanisms: First, high concentrations of Ca^2^ ⁺ ions lead to an increase in osmotic pressure, which compromises the stability of the cell membrane. Elevated osmotic pressure or ion strength interferes with the structure and function of urease or affects bacterial metabolic processes. Second, high pH levels or calcium salt crystallization processes induce unfavorable changes in the microenvironment, thereby inhibiting microbial proliferation and urease expression. It is evident that the concentration of the cementation solution not only regulates the donor ion level for calcium carbonate precipitation but also exerts a feedback effect on microbial metabolism. Based on a comprehensive analysis of microbial survival rates and urease activity, 0.5 mol/L can be considered as the optimal balance point between microbial activity and cementation efficiency in this experiment [55–61].

### Penetration test result analysis

It can be discerned from Table 6 that when the concentration of the cementation solution is 0.5 mol/L, the permeability coefficient of the sample is relatively low; when the concentration of the cementation solution is 0.2 mol/L, the permeability coefficient of the sample is higher; when the concentration of the cementation solution rises from 0.2 mol/L to 0.4 mol/L, the permeability coefficient of the sample gradually diminishes. When the concentration of the cementation solution exceeds 0.5 mol/L, the permeability coefficient of the sample gradually ascends. When the concentration of the cementation solution is 0.5 mol/L, the standard deviation is relatively small at 0.08 x10^-4^, and the permeability coefficient is also relatively low. This indicates that the internal structure of the sample is relatively compact under these conditions. When the concentration of the cementation solution ranges from 0.2 to 0.4 mol/L, the standard deviation gradually decreases, with values of 0.23 x10^-4^, 0.19 x10^-4^, and 0.16 x10^-4^, respectively. The permeability coefficient also decreases progressively, suggesting an increasing content of calcium carbonate crystals, which gradually fill the interior of the sample. When the concentration of the cementation solution exceeds 0.6 mol/L, the standard deviation begins to increase again, with values of 0.14 x10^-4^ and 0.15 x10^-4^ for the respective samples. This indicates that urease activity is inhibited under these conditions, leading to a gradual decrease in the content of calcium carbonate crystals, an increase in pore volume within the samples, and a corresponding rise in the permeability coefficient.

**Table 6:** Permeability coefficients of 6 different cementation solution concentrations.

cementation solution concentrations(mol/L)	Permeability Coefficient(cm/s)	cementation solution concentrations(mol/L)	Permeability Coefficient(cm/s)
0.2	6.8x10^-4^	0.3	5.9x10^-4^
0.4	3.7x10^-4^	0.5	1.8x10^-4^
0.6	1.9x10^-4^	0.7	2.1x10^-4^

The permeability data in Table 6 reveal a nonlinear trend, with the 0.6 mol/L cementation solution exhibiting slightly higher permeability (1.9 × 10 ⁻ ⁴ cm/s) compared to 0.5 mol/L (1.8 × 10 ⁻ ⁴ cm/s). This deviation stems from a complex interplay of factors influencing calcite precipitation dynamics. At higher concentrations (≥0.6 mol/L), enzyme inhibition occurs as elevated calcium ion levels suppress urease activity, reducing CaCO₃ deposition efficiency and compromising pore-filling uniformity. Additionally, rapid precipitation promotes the formation of larger, looser CaCO₃ aggregates, diminishing structural density, while spatial heterogeneity leads to localized clogging that leaves deeper pores untreated. In contrast, the 0.5 mol/L concentration facilitates gradual, uniform precipitation through slower reaction kinetics, enabling deeper diffusion and more effective pore bridging. Microbial constraints further contribute to this nonlinearity, as osmotic stress at 0.6 mol/L impedes bacterial mobility, restricting homogeneous calcite distribution. Although the permeability difference is marginal, the 0.5 mol/L concentration demonstrates superior overall performance by balancing microbial viability, precipitation uniformity, and mechanical strength. This highlights that permeability is governed not only by calcite quantity but also by its spatial distribution, sedimentation kinetics, and microbial dynamics, underscoring the need for optimized concentrations to achieve maximal permeability reduction through deep, uniform treatment.

It can be observed from Table 7, as the concentration of the cementation solution increases from 0.2 mol/L to 0.4 mol/L, the standard deviation exhibits a gradual decrease. Upon reaching a concentration of 0.5 mol/L, the standard deviation becomes relatively minimized. However, when the concentration exceeds 0.5 mol/L, the standard deviation demonstrates a gradual increase. As presented in Table 5, with the concentration of the cementation solution increasing from 0.2 mol/L to 0.4 mol/L, the standard deviation demonstrates a consistent downward trend. Upon reaching a concentration of 0.5 mol/L, the standard deviation attains its minimum value. Conversely, as the concentration surpasses 0.5 mol/L, the standard deviation starts to exhibit a gradual upward trend. The calcium carbonate precipitation produced by MICP technology fills the pores of the sample, augmenting its compactness and resistance to permeability, thereby reducing the permeability coefficient. Consequently, when the concentration of the cementation solution gradually increases to an appropriate extent, the microbial reaction is relatively rapid, leading to a large number of calcium carbonate crystals being filled between sand particles. Nevertheless, when the concentration of the cementation solution is excessively high, it will affect the microbial mineralization process, thus influencing the generation of calcium carbonate crystals and the filling of the internal pores of the sample, and subsequently increasing the permeability coefficient. In the sandy environment, a judicious reduction of the permeability coefficient is conducive to water retention and the conservation of desert green plants, thereby exerting a significant role in environmental protection.

**Table 7:** Standard deviation of Permeability coefficients in 6 different cementation solution concentrations.

Cementation solution concentration (mol/L)	Permeability coefficients (cm/s)	Standard deviation(cm/s)
0.2	6.8x10^-4^	0.62x10^-4^
0.3	5.9x10^-4^	0.51x10^-4^
0.4	3.7x10^-4^	0.39 x10^-4^
0.5	1.8x10^-4^	0.13 x10^-4^
0.6	1.9x10^-4^	0.18 x10^-4^
0.7	2.1x10^-4^	0.21 x10^-4^

### Nuclear magnetic resonance test results analysis

**Fig 19:**
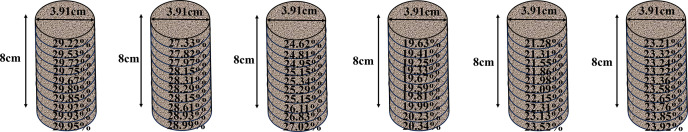
Porosity at different positions of 6 samples with different cementation solution concentrations.

**Fig 20:**
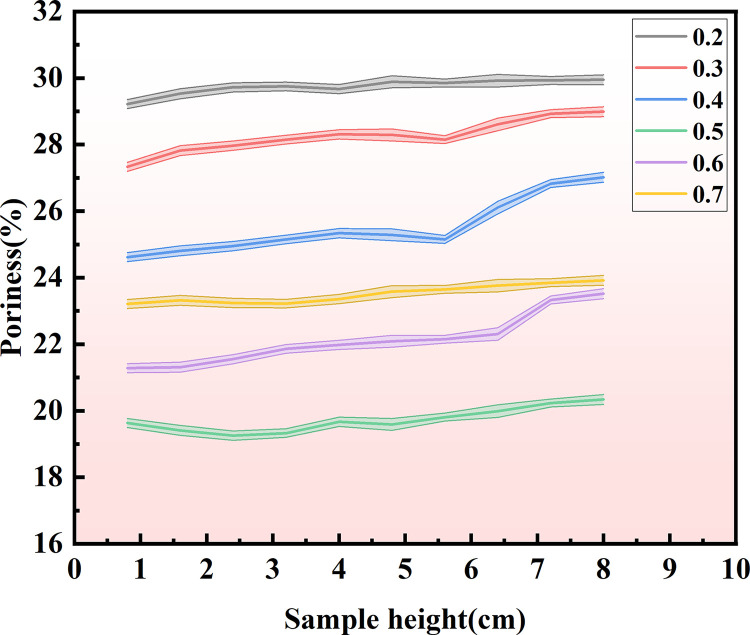
Porosity curves of 6 different cementation solution concentrations at different locations.

It is evident from Fig 19. that when the concentration of the cementation solution amounts to 0.5 mol/L, the porosity of the sample is relatively low; when the concentration of the cementation solution is 0.2 mol/L, the porosity of the sample is higher; when the concentration of the cementation solution rises from 0.2 mol/L to 0.4 mol/L, the porosity of the sample gradually diminishes. When the concentration of the cementation solution exceeds 0.5 mol/L, the porosity of the sample gradually ascends. As depicted in Fig 20, the porosity of samples with varying concentrations of the cementation solution fluctuates in each part. This is ascribed to the non-uniform formation of calcium carbonate crystals during the microbial solidification process, giving rise to different microbial mineralization conditions in different parts, which also constitutes a challenge for future research breakthroughs. Furthermore, reducing the porosity of sand can be utilized in some straightforward seepage prevention projects to effectively prevent the loss of sand.

The observed fluctuations in porosity percentage (%) across different sample positions in the NMR data, despite uniform experimental conditions, can be attributed to several interrelated factors. Firstly, the limited diffusion of microorganisms in sandy soil may lead to localized enrichment or depletion, affecting calcium carbonate precipitation efficiency. Secondly, the flow paths of bacterial and cementation solution are influenced by the microscopic pore structure and initial density, causing regional variations in reactant concentrations. Thirdly, uneven deposition rates of cementation products in certain areas further exacerbate porosity differences. Additionally, the non-uniform distribution and aggregation of palm fibers play a significant role: random fiber dispersion creates dense regions that act as physical barriers, impeding fluid penetration and resulting in high-porosity zones, whereas sparse fiber areas exhibit lower porosity as a result of more complete reactions. Fiber orientation also contributes, as directional arrangements induce anisotropic permeability, leading to porosity variations along the fiber axis. Furthermore, local microbial accumulation or absence--caused by fiber blockage or nutrient competition-can lead to non-uniform calcium carbonate precipitation. Lastly, inconsistencies in sample preparation, such as uneven compaction, introduce initial pore distribution heterogeneity, which subsequently affects the homogeneity of the MICP process. Together, these factors explain the spatial porosity variations observed in the sample.

### Calcium carbonate content analysis

As shown in Table 8, when the concentration of the cementation solution reaches 0.5 mol/L, the sample exhibits a relatively high content of calcium carbonate with a correspondingly small standard deviation. At a cementation solution concentration of 0.2 mol/L, the calcium carbonate content is comparatively lower. As the concentration increases from 0.2 mol/L to 0.4 mol/L, the calcium carbonate content in the sample gradually rises while the standard deviation progressively diminishes. Conversely, when the concentration exceeds 0.5 mol/L, the calcium carbonate content decreases and the standard deviation increases. For concentrations below 0.5 mol/L, an increase in the concentration of the cementation solution leads to a higher availability of active substances in the solution, including urea and calcium ions, thereby promoting the deposition of more calcium carbonate crystals. When the concentration of the cementation solution is high (exceeding 0.5 mol/L), the high concentration of the cementation solution exerts a certain inhibitory effect on microorganisms, resulting in a reduced amount of urease production and a decrease in the content of calcium carbonate. It is generally recognized [53–62] that calcium carbonate content serves as an important indicator for evaluating the cementation and curing effect of MICP, and a higher calcium carbonate content constitutes the primary source of the enhanced strength of MICP-solidified sand. On the one hand, calcium carbonate precipitation can fill the pores of sand and bind sand particles together. When the sandy soil is subjected to shear force, it can more effectively resist the displacement interleaving between the sand particles to enhance the strength of the sand.

**Table 8:** Calcium carbonate content and Standard deviation in 6 different cementation solution concentrations.

cementation solution concentrations(mol/L)	0.2	0.3	0.4	0.5	0.6	0.7
Calcium carbonate content (%)	7.3	9.7	12.3	15.8	14.1	13.6
Standard deviation (%)	0.92	0.87	0.83	0.36	0.69	0.71

The data in Table 8 reveals a nonlinear relationship between CaCO₃ content and unconfined compressive strength, which can be attributed to the complex interplay of microbial activity, crystallization dynamics, and reaction conditions. Although the CaCO₃ content at 0.6 mol/L (14.1%) is slightly higher than that at 0.7 mol/L (13.6%), the corresponding the unconfined compressive strength values (582 kPa vs. 558 kPa) do not follow a strict linear trend. This discrepancy arises because higher concentrations (e.g., 0.7 mol/L) may inhibit microbial activity as a result of osmotic stress, reducing urease production and leading to uneven CaCO₃ distribution. Additionally, accelerated precipitation at elevated concentrations can result in CaCO₃ primarily filling pores rather than forming effective bridging structures at particle contacts, thereby diminishing strength enhancement despite similar or slightly lower carbonate content. Furthermore, rapid pore clogging at higher concentrations shortens the reaction window, limiting sustained and uniform cementation. In contrast, the 0.6 mol/L concentration optimizes microbial metabolic efficiency, promotes favorable crystal morphology, and ensures a more homogeneous distribution of CaCO₃, ultimately yielding higher strength. This demonstrates that the unconfined compressive strength depends not only on the total CaCO₃ content but also on its spatial distribution, crystallization patterns, and the synergistic effects of biochemical and physical factors during the cementation process.

## Microanalysis

**Fig 21:**
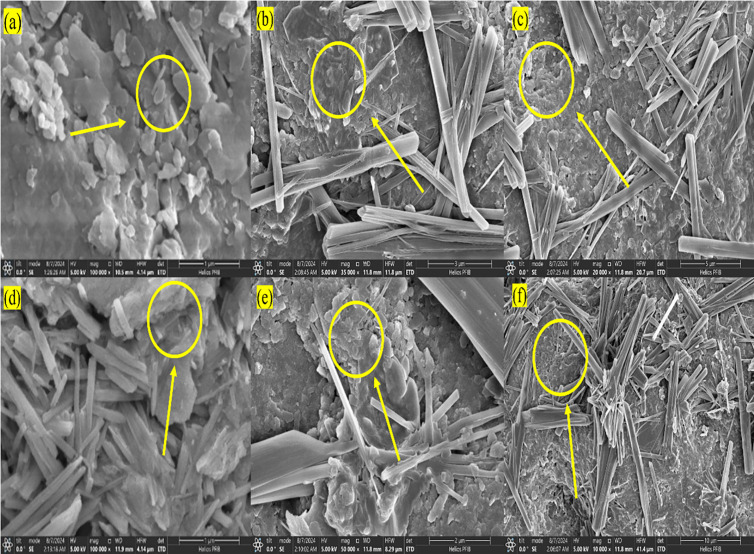
SEM images of cementation solution concentrations of (a) 0.2 mol/L, (b)0.3 mol/L, (c) 0.4 mol/L, (d) 0.5 mol/L, (e) 0.6 mol/L and (f) 0.7 mol/L respectively.

From Fig 21, it is evident that the generated calcium carbonate crystals fill the original pores between sand grains to a certain extent. Fig 21(a) illustrates the scanning electron microscopy (SEM) image of the cured sample with a cementation solution concentration of 0.2 mol/L. Notably, only a few calcium carbonate crystals are present on the fiber surfaces, and no significant precipitation is observed either on or near the fiber surfaces. Fig 21(d) depicts the SEM image of the solidified sample at a concentration of 0.5 mol/L, where a greater number of calcium carbonate crystals are clearly visible near the fibers. In contrast, Fig 21(f) shows the SEM image of the solidified sample at a concentration of 0.7 mol/L, revealing that the formation of calcium carbonate crystals near the fibers is less favorable compared to that in Fig 21(d). For low concentrations Fig 21(a-c): The crystal size is relatively small, with pronounced agglomeration, likely consisting primarily of amorphous spherical particles. The crystal structure exhibits an amorphous or microcrystalline aggregation state, characterized by blurred boundaries and small particle sizes. This phenomenon may arise from insufficient ion supersaturation at low concentrations, resulting in weak crystallization driving forces and incomplete crystal forms. For suitable concentrations Fig 21(d): Large-sized calcite (regular rhombohedron) dominates, featuring smooth crystal surfaces, highly regular crystal morphology, prominent rhombohedral columnar structures of calcite, and distinct edges and corners. The increase in intercrystal porosity indicates an adequate supply of ions at this concentration. For high concentrations Fig 21(e-f): Excessive supersaturation may induce heterogeneous nucleation bursts, leading to the formation of numerous tiny crystal nuclei that compete for limited ionic resources, ultimately generating crystals with uneven sizes and many defects (such as twins and dislocations). Irregular aggregates are observed in the SEM images instead of single regular crystal forms.

This can be attributed to the fact that, as the concentration of the cementation solution increases, the morphology of calcium carbonate crystals becomes increasingly pronounced. In Fig 21(a-c), the calcium carbonate crystals are characterized by a small volume and limited quantity. The weak inter-particle bonding between sand grains and the presence of numerous pores suggest that this type of calcium carbonate crystalline precipitation may not possess sufficient shear strength. As a result, the curing effect of low-concentration cementation solutions is less than optimal. In contrast, in Fig 21(d), the number of calcium carbonate crystals on the surface of sand grains and near fibers markedly increases, with their volume expanding, clustering becoming more evident, and inter-particle cementation becoming more compact. At this concentration, the cementation solution provides an adequate supply of Ca^2^⁺ and CO_3_^2^ ⁻ ions, which promotes directional crystal growth following nucleation and facilitates the formation of a more complete lattice structure. This observation is directly supported by the trend toward regularity in crystal size and morphology observed in SEM images. For samples cured with high-concentration cementation solutions (Fig 21(e-f)), the calcium carbonate crystals tend to be larger in size and predominantly exhibit layered and blocky forms. However, as the concentration of the cementation solution increases, the lower portion of the sample becomes more susceptible to pore blockage, resulting in a less prominent overall curing effect and only marginal improvements in unconfined compressive strength.

**Fig 22:**
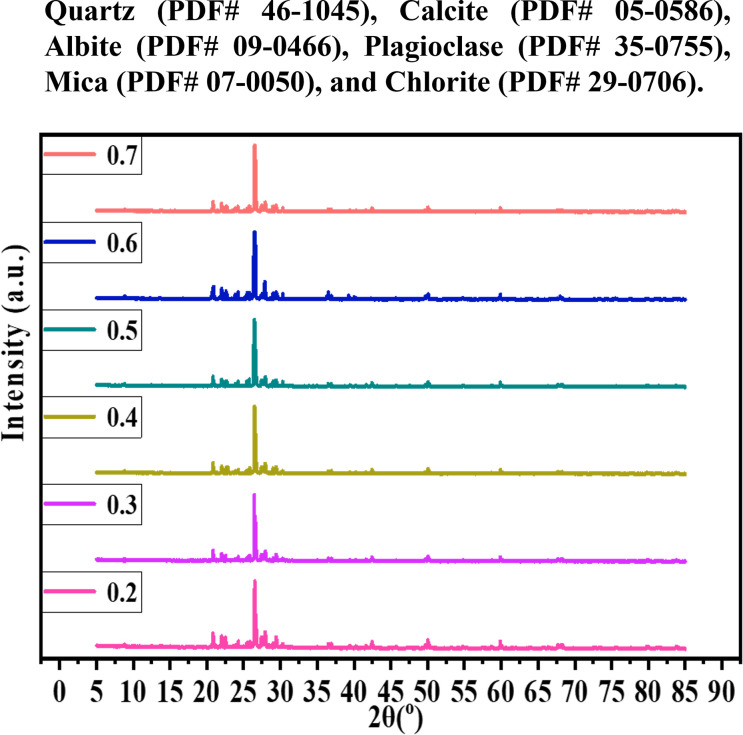
The XRD results of cementation solution concentrations of 0.2 mol/L-0.7 mol/L were obtained.

The XRD test outcomes are depicted in Fig 22. It can be discerned that the principal mineral of sandy soil is quartz. Nevertheless, subsequent to microbial solidification, a characteristic diffraction peak of calcium carbonate is also present in the soil sample, whose main crystal form is calcite and whose dominant crystal face is (40), thereby demonstrating that the calcium carbonate generated by microbial solidification in sandy soil is the direct reason for its strength enhancement. Furthermore, the peak strength of the cementation solution with a concentration of 0.5 mol/L was markedly higher than that of the other two types of cementation solution. This is predominantly because the calcium carbonate content of the sample with a concentration of 0.5 mol/L rose, and its crystalline phase content increased, resulting in the decrease of the calcium carbonate crystalline phase content and corresponding peak strength of the samples with the other two concentrations. The mineral analysis results suggest that the calcium carbonate crystals obtained through MICP curing are stable calcite crystals. The concentration of the cementation solution is a crucial factor affecting the formation of calcium carbonate crystals, and a rational concentration of the cementation solution can guarantee the optimal precipitation of calcium carbonate, which is highly significant for the curing effect.

The intensity of the main calcite peak at each concentration, typically attributed to the (104) crystal face corresponding to 2θ ≈ 29.4°, represents one of the most prominent peaks. The remaining calcite peaks are distributed approximately at 2θ = 23°, 35°, 39°, 47°, etc. The intensities of the seven peak groups were normalized, with the maximum value set to 1.

It can be observed from Table 9 that X-ray diffraction (XRD) analysis was performed on MICP samples treated with varying concentrations (0.2–0.7 mol/L) of cementation solution. The intensity of the main calcite peak at 2θ ≈ 29.4° was selected as the quantitative indicator. Following normalization (based on the maximum main peak intensity), the results demonstrated that as the concentration of the cementation solution increased from 0.2 mol/L to 0.5 mol/L, the calcite content exhibited a significant increase, with the normalized peak intensity rising from 0.867 to 1.000. This indicates that the highest amount of calcite was generated at a cementation solution concentration of 0.5 mol/L. When the cementation solution concentration was elevated to 0.7 mol/L, the calcite content began to decrease gradually, and the normalized peak intensity dropped from 1.000 to 0.948. This trend aligns with the findings from mechanical property tests, further validating that 0.5 mol/L is the optimal concentration of the cementation solution in this study.

**Table 9:** Normalized intensity.

cementation solution concentration (mol/L)	Intensity (a.u.)	Normalized intensity (maximum value = 1)
0.2	23200	23200/26700 ≈ 0.867
0.3	23700	23700/ 26700 ≈ 0.887
0.4	24800	24800/ 26700 ≈ 0.929
0.5	26700	26700/ 26700 = 1.000
0.6	25800	25800/26700 ≈ 0.966
0.7	25300	25300/26700 ≈ 0.948

## Discussion

Currently, most studies on MICP for sand fixation have primarily focused on strain selection, injection techniques, and precipitation mechanisms [63,64]. However, regarding cementation solution concentration, most studies have only verified 1−2 concentration conditions, lacking systematic comparisons [65] utilized only 0.5 mol/L for sand reinforcement). Moreover, most existing studies [66,67] have not incorporated natural fiber materials, leading to common issues such as high brittleness and poor durability in consolidated bodies. This study advances MICP-based reinforcement by systematically investigating a gradient of cementation solution concentrations (0.2–0.7 mol/L), establishing a quantitative microstructure-mechanical property relationship for palm fiber-calcium carbonate composites to overcome pore clogging (at high concentrations) or inadequate strength (at low concentrations). Key innovations include: (1) revealing the nonlinear impact of concentration on MICP performance, (2) introducing renewable palm fibers to enhance toughness, (3) proposing an “optimal concentration interval” by integrating mechanical, permeability, and porosity analyses, and (4) combining natural fibers with green microbial mineralization to improve eco-friendliness and functionality. These breakthroughs address prior research gaps, optimizing MICP sand fixation for enhanced stability, ecological sustainability, and engineering applicability.

The 0.5 mol/L cementation solution concentration optimizes microbial activity, solution saturation, and calcite crystallization to maximize the strength of MICP-reinforced soil by forming an optimal coupling point between mechanical properties and biological reaction efficiency. This concentration provides a moderate osmotic pressure environment that sustains bacterial viability and urease stability while preventing salt-induced inhibition, ensuring uniform microbial distribution and sustained urea hydrolysis. The error ranges across different concentrations also imply that overly high or low solution molarities may induce uneven microbial activity or Ca^2^ ⁺ diffusion, affecting reproducibility. Additionally, it maintains an ideal saturation of Ca^2^⁺ and urea, enabling a controlled precipitation rate of CaCO₃ that avoids uneven deposition and enhances interparticle cementation through well-formed bridging structures [68–70]. Furthermore, the 0.5 mol/L condition favors the stable growth of dense, regular calcite crystals-which exhibit superior thermodynamic stability and mechanical strength compared to other polymorphs-while minimizing loose or irregular crystal formation. Collectively, these mechanisms synergistically enhance the structural integrity and unconfined compressive strength of the treated soil.

Recent studies have demonstrated that fiber reinforcement significantly improves the toughness of MICP-treated soils compared to non-fiber MICP. Liang [71] investigated the influence of fiber type and length on MICP-treated sand, revealing that fibers notably enhance tensile strength and fracture toughness, particularly with short fibers at optimal content. Similarly, Liu [72] found that basalt fiber reinforcement (BFR) in MICP-stabilized aeolian sand improves unconfined compressive strength and crack resistance, with fiber length and dosage playing critical roles. Wang [73] further highlighted that fiber reinforcement, combined with optimized microbial culture conditions, enhances both strength and toughness in MICP-stabilized soils. Comparative data indicate that non-fiber MICP exhibits low tensile strength (relatively low values) and brittle failure, with elongation rates of 0.15–0.18, whereas fiber-reinforced MICP achieves higher elongation (0.19–0.27) and tensile strength, along with restricted crack propagation, leading to a 20–40% increase in toughness. This improvement is attributed to fiber bridging and crack-arresting mechanisms, making fiber-reinforced MICP superior for applications requiring ductility and crack resistance, such as ground improvement and environmental remediation.

## Conclusion

Palm fiber-reinforced MICP technology represents an environmentally friendly and sustainable solution for soil reinforcement, attracting considerable attention in ecological sand stabilization, soil and water conservation, and surface stability engineering. This study systematically investigates the effects of varying cementation solution concentrations (0.2–0.7 mol/L) on the mechanical properties and permeability of MICP-reinforced sand samples to evaluate their potential applications in environmental engineering. The results demonstrate that a concentration of 0.5 mol/L achieves the highest unconfined compressive strength (666.65 MPa), significantly outperforming both lower concentrations (e.g., 0.2 mol/L, 278.59 MPa, 0.3 mol/L, 379 MPa) and higher concentrations (e.g., 0.6 mol/L, 582.63 MPa, 0.7 mol/L, 558.12 MPa). Additionally, at low concentrations (0.2–0.4 mol/L), shear stress increases progressively, while the permeability coefficient and porosity decrease gradually. In contrast, as the concentration rises from 0.5 mol/L to 0.7 mol/L, shear stress decreases, permeability coefficient and porosity increase, and both mechanical properties and microbial activities decline. These findings indicate that high cementation solution concentrations inhibit bacterial physiological functions and reduce calcium carbonate deposition efficiency, thereby limiting the overall performance of MICP-reinforced sand [74,75].

This indicates that at low concentrations, the limited deposition of calcium carbonate constrains the consolidation effect. In contrast, although high concentrations supply sufficient cementitious materials, they suppress microbial enzyme activity, thereby hindering the formation of uniform crystals and the improvement of mechanical strength. Future studies should focus on optimizing the concentration near 0.5 mol/L to explore the synergistic effects between fiber dosage and environmental factors. The engineering feasibility of MICP technology for sand fixation and soil improvement can be enhanced by fine-tuning the concentration gradient (e.g., 0.45 mol/L and 0.55 mol/L) to accurately balance microbial activity and solidification reactions, or by combining biological regulation strategies with composite cement agents to alleviate microbial inhibition at high concentrations. This highlights that the optimal cementation solution concentration promotes the development of a uniform and stable cementing structure, where calcium carbonate precipitation fills soil pores, significantly reduces permeability, decreases total porosity, and constricts water flow channels. These changes enhance water retention capacity through increased micropore density, improved capillary water absorption, and reduced evaporation, while a denser surface layer improves erosion resistance and mechanical stability.

## Practical implications and Limitations

MICP technology holds promise for large-scale applications such as sand fixation, but its widespread adoption faces critical challenges related to cost, bacterial viability, and long-term durability. The high cost of cementation solutions (urea and calcium salts) remains a major barrier, as optimizing concentration to balance strength and microbial activity without escalating expenses is complex. Additionally, extreme desert conditions-high temperatures, low humidity, and UV radiation-severely hinder bacterial survival and enzymatic efficiency, necessitating the development of resilient microbial strains and rapid on-site cultivation methods [76–81]. Furthermore, the long-term stability of MICP-treated structures is uncertain, as wind erosion, rain, and UV exposure can degrade both the calcium carbonate matrix and organic reinforcements like palm fibers. Current research focuses on three key areas: (1) formulating low-cost, high-efficiency cementation systems, (2) screening robust bacterial strains and refining cultivation techniques, and (3) enhancing fiber modification to improve environmental resistance. Addressing these challenges systematically is essential to advance MICP toward scalable, sustainable engineering applications.

### Practical implications

Palm fiber-reinforced MICP sand fixation technology has demonstrated significant potential in environmental and geotechnical engineering. Its primary applications include:

Windbreak and Sand Fixation: A moderate cementation solution concentration (e.g., 0.5 mol/L) enhances sand cohesion and wind erosion resistance, making it suitable for arid and semi-arid regions.

Soil and Water Conservation: Low to medium concentrations (0.2–0.5 mol/L) improve soil structure and water retention while maintaining microbial activity, aiding ecological restoration.

Engineering Reinforcement: High-concentration solutions (0.6–0.7 mol/L) provide strong mechanical stabilization for foundations and subgrades, though cost and environmental trade-offs must be considered.

### Limitations

Despite its advantages, the technology faces several challenges:

High Costs: Large-scale use of high-concentration cementation solutions is economically restrictive.

Reduced Bacterial Efficiency: Concentrations above 0.6 mol/L can inhibit microbial activity, leading to uneven consolidation.

Environmental Degradation: Palm fibers may degrade over time because of moisture, UV exposure, and microbial action, reducing long-term effectiveness.Construction Complexity: Variations in cementation solution requirements increase operational difficulty and consolidation inconsistency.

Ecological Risks: Excessive cementation solutions may alter soil chemistry and harm microbial and plant life, necessitating ecological assessments.

Future improvements should focus on optimizing material ratios, enhancing microbial efficiency, and ensuring environmental sustainability.

## Supporting information

S1 File Shear Displacement-Shear Stress Curves Data of 6 Different Cementation Solution Concentration and Stress-Strain Curves Data of 6 Samples with Different Cementation Solution Concentrations. (XLSX)
